# Insights into Children with Down Syndrome: A Medical Student’s Perspective

**DOI:** 10.31729/jnma.8244

**Published:** 2023-08-31

**Authors:** Aashis Poudel

**Affiliations:** 1 Patan Academy of Health Sciences, Lagankhel, Lalitpur, Nepal

**Keywords:** *developmental disabilities*, *Down syndrome*, *holistic health*, *medical student*

## Abstract

Down syndrome is the most common chromosomal abnormality among liveborn infants that frequently causes intellectual disability. However, with proper medical care and support, children with Down syndrome can still lead fulfilling lives and achieve their full potential. The experience at Satyam Day Care Center has provided valuable insights into the challenges and opportunities of caring for children with Down syndrome. Advocating for increased awareness and understanding of Down syndrome, including its genetic causes, associated health conditions, and developmental delays is important.

## INTRODUCTION

Down syndrome is the most common chromosomal abnormality among liveborn infants that frequently causes intellectual disability.^1^ Around one in every 800 babies are born with Down syndrome globally.^2^ Majority of individuals have complete extra chromosome 21 as a result of nondisjunction during first meiotic division.^3^ People with Down syndrome have a wide array of disorders: neuropsychiatric disorders, cardiovascular disease, gastrointestinal abnormalities, endocrine disorders, haematological disorders, and immunodeficiencies. These problems grow exponentially to result in delays in development.^1,4^ However, with early intervention programs and therapies, people with Down syndrome can still lead fulfilling lives, achieve their full potential and prevent associated complications.

## EXPERIENCE

Given the statistical prevalence of babies being born with Down syndrome, it occurred to me that I had not personally witnessed any children with Down syndrome attending school or going to job. This made me curious to inquire about the education and occupational opportunities available to individuals with Down syndrome. I was also interested in understanding how these children thrived in their daily lives, whether they lived with their families or in specialized care centres, and how they contributed to the workforce as they transitioned into adulthood.

I came across Global Downs Syndrome Foundation under which at least one organization worked in each country.^5^ That is how I came across Satyam Day Care Center which was under the Down's Syndrome Society of Nepal (DSSN). This organization works on providing holistic care to the children for their overall development which allows them to live independently and work for themselves.

Before visiting the centre, my perception was that I would primarily encounter school-aged children with Down syndrome. However, I was surprised to meet individuals: majority of the whom were adults. This shows the importance of recognizing and addressing the needs of individuals with Down syndrome across the lifespan. There were 18 students ranging from age 5 to 36 years and 3 teachers looking after them in the daycare centre. Although I was a new face to them, I was greeted every day and they shared their problems with me.

I had no interaction with individuals having Down syndrome before. I had studied that they have characteristic physical features such as a flat face with a smaller nasal bridge, upward-slanting eyes with epicanthic folds, low-set ears, and short stature. However, upon engaging with them, I realized although these physical features often serve as distinguishing characteristics, it does not define the entirety of the condition. Instead, a key characteristic that distinguished the Down syndrome individuals at DSSN was developmental delay. This brings light to the multifaceted nature of Down syndrome, which extends beyond its physical manifestations.

## ACTIVITIES AT THE CENTRE

I observed the daily activities of the children and focused on how care was provided in the centre. The teachers there used various strategies and unique styles for supporting their learning and communication. Education is equally important for all so the children were thoughtfully separated into two groups based on their abilities. Individuals who lacked adequate motor and linguistic abilities were kept in one group, while others were kept in a different group. Each group set a distinct goal for reaching milestones. The individuals in the second group were more concerned with social achievements. This learning approach encouraged mutual support and growth among peers. This allowed the students to learn effectively and fostered a sense of friendship and inclusivity within the classroom, creating an emotionally supportive and nurturing environment for all. In addition to their academic activities, they did morning exercises, danced, sang bhajans, played musical instruments and indoor games. These activities were beneficial for their physical fitness and improved their emotional and mental well-being.

## LEARNING POINTS

I enjoyed talking to and helping patients with either academics or practical aspects of life. In the morning assembly, they used to dance and do physical exercises. It was challenging for me to understand what they were saying initially, but the teachers there helped me all the way. I even assessed some domains of the developmental milestones in these children. I used to think these children have developmental delays and they could not learn anything, but after my time in the centre, I realized that children with Down syndrome can achieve milestones with patience, dedication, and support. The teachers at the centre worked tirelessly for the betterment of the patients there. I used to think that children with Downs syndrome were incapable of learning, working, or living independently but seeing them, and talking to their mother made me realize that they are capable of learning, working, and contributing to society in meaningful ways. Despite positive results in younger participants, the older members of the group functional decline and increased concern for their families.

## IMPORTANCE OF EARLY INTERVENTION AND EDUCATION

It is essential to know about the genetic cause, prenatal diagnosis, associated health conditions of the child and developmental delay that may be present in children with Downs syndrome. The earlier people know about this condition, the earlier the health and developmental intervention can be made, which will benefit them.

## IMPACT ON PARENTS AND FAMILIES

While talking with the parents about Downs syndrome, they told me their perspective on it and its impact on their family. Some parents shared feelings of stress, anxiety, and isolation, and struggle to find support from others, especially from the school their children used to study before. Other siblings were affected, as they had to adjust with limited time and had their own emotional needs. Many parents shared that they did not know about the prenatal diagnosis of Downs syndrome. In Nepal, many parents do not have access to information regarding health conditions, prenatal diagnosis and developmental issues which makes them unable to provide their children with the support they need.^6^ While other parents thought that the impact of Down syndrome on their families was positive, as children with Down syndrome brought joy, love, and a unique perspective to their families.

Staying there for 2 weeks made me realize it needed a great deal of patience and creativity to teach them, and I have a lot to work on my communication skills. Interacting and engaging with these people was incredibly rewarding and fulfilling. I developed an increased understanding and awareness of disability, as well as the social, emotional, and practical challenges that people with disabilities and their families may face.

## WAY FORWARD

The experience at Satyam Day Care Centre has provided valuable insights into the challenges and opportunities of caring for children with Down syndrome. Going forward, it is important to advocate for increased awareness and understanding of Down syndrome, including its genetic causes, associated health conditions, and developmental delays. The focus should be wider than the pathophysiology and clinical features. We must emphasize developing effective strategies for supporting their learning and communication. Communicating with adults is easier than with children. So, as a medical student, I realized that to better grasp this skill we need to spend time with children in the pediatric ward or pediatric referral clinic and try to build a rapport and get necessary information with them rather than their informant. Providing access to education and learning environments can also help them develop their strengths, overcome challenges, and form meaningful connections with others. As volunteers, we have the privilege of witnessing a nurturing environment that promotes holistic development and well-being.

Through active engagement with individuals with Down syndrome, we can contribute to research, advance our understanding of their unique abilities and cultivate valuable skills in communication, patience, and adaptability. Additionally, supporting parents and families of children with Down syndrome through counselling and education can help them better understand and navigate the social, emotional, and practical challenges they may face.
